# Editorial: Geomicrobes: Life in Terrestrial Deep Subsurface

**DOI:** 10.3389/fmicb.2017.00103

**Published:** 2017-01-31

**Authors:** Malin Bomberg, Lasse Ahonen

**Affiliations:** ^1^VTT Technical Research Centre of Finland Ltd.Espoo, Finland; ^2^Geological Survey of FinlandEspoo, Finland

**Keywords:** deep biosphere, iron oxidation, heavy metal resistance, bedrock aquifer, limestone cave, mine microbiology, biogeological methane cycling

## Introduction

The deep terrestrial biosphere is an intriguing research field linking to astrobiology and evolution of life on early Earth (Grosch and Hazen, 2015). Living in the deep, dark, anoxic, oligotrophic, saline, highly pressurized and often hot subsurface requires some striking characteristics of the inhabitants (Kieft, 2016). We still know only little about the biochemical processes actually taking place deep in the Earth's crust, or about the interactions within microbial communities residing in the isolated aquifers. Nevertheless, the deep subsurface is planned to serve as geological repository for e.g., nuclear and other hazardous wastes and carbon dioxide (De Coninck and Benson, 2014; Russell et al., 2015). We still do not know how the long isolated deep bedrock environments will respond to these intrusions, or what the fate of the repositories will be. With this Research Topic we aimed to collect new knowledge about the roles and functions of microbial communities in the undisturbed or disturbed subsurface and called upon our colleagues to step up to the task. The deep subsurface is of interest worldwide and we received contributions from Asia, Australia, Africa, North America, and Europe!

## What did we learn?

The deep terrestrial subsurface is difficult to access, but can be reached through drillholes in the rock that reach the aquifers (Sohlberg et al.), cave streams (Brannen-Donnely and Engel), water seeping through mine walls (Borgonie et al.), drillholes in mines and caves (Miettinen et al.; Rajala et al.; Bonis and Gralnick; Kutvonen et al.) and by collecting rock material and scrapings from caves (Wu et al.) and mines (Dziewit et al.). The deep subsurface microbial communities are very diverse, spanning all domains of life (Borgonie et al.; Miettinen et al.; Sohlberg et al.).

Metals are important factors driving deep subsurface life (e.g., Parnell et al., 2016). Iron, one of the most common compounds in the Earth's crust, plays an important role and is part of many biomolecules. Reduced iron may serve as electron source for iron-oxidizing microorganisms, such as *Marinobacter subterrani* (Bonis and Gralnick) living in the iron-rich calcium chloride brine in the Soudan iron mine at more than 700 m depth. Iron redox reactions may support vast microbial communities in various environments. Iron oxidation occurs in many microbial species, which may affect the corrosion of e.g., carbon steel in deep geological repositories for radioactive waste. Radioactive metallic wastes originate from decommissioning activities in nuclear power plants. In Olkiluoto nuclear power plant, Finland, the decommissioning wastes are stored in an underground bedrock repository at 60–100 m depth. The temperature of the surrounding groundwater affects the communities causing microbially induced corrosion of carbon steel.

In the Lubin copper mine, Poland, microorganisms are exposed to high levels of heavy metals (HM) and diverse resistance strategies are employed. Dziewit et al. isolated HM resistant bacteria from the mine and found several plasmids and broad-host-range transposons carrying various HM resistance markers. HM tolerance genes located on extra chromosomal elements can play a key role in the adaptation of microbial communities to HM stress, due to possible horizontal gene transfer.

Pyhäsalmi mine in Finland, one of Europe's deepest mines, provides an opportunity for sampling deep groundwater fractions through underground drillholes reaching more than 2 km below ground. Only low numbers of microbial cells were found in these deep, saline and high-pressure fluids (Miettinen et al.). However, the microbial diversity was surprisingly high, including bacteria, archaea and fungi. Borgonie et al. managed to reach even deeper, down to 3.4 km depth, in the South African Tona Tau gold mine. Sampled stalactites revealed life forms, even nematodes, trapped in the stalactite since its formation, displaying a signature for the origin of the deep groundwater and earlier conditions on Earth.

In an epigenic carst stream with fast turnover rates and seasonal variations in water volumes and nutrient flow, Brannen-Donnelly and Engel showed microbial lineages that were specific to the different stream habitats but did not appear between the habitats (running water or static sediment). Rajala et al. reported similar results as biofilm on carbon steel surfaces differed distinctly from the planktonic community in Fennoscandian groundwater. Wu et al. showed that the bacterial community colonizing the walls of the limestone Jinjia Cave, China, changed with growing distance from the cave mouth. The authors suggested that the deepest end of the cave was inhabited by obligate cave-colonizing Gammaproteobacteria often detected from caves world-wide.

Nitrogen compounds, such as nitrate and ammonia, are generally in short supply in deep groundwater, but may enter the depths through explosives used in the bedrock or through infiltration from the surface. The bacterial numbers increased significantly when these compounds were available and radically changed the microbial community structure (Kutvonen et al.). The bacteria that used these compounds as energy source were distinct from those using them as nitrogen source. Many of the bacteria detected were methylotrophs, which may be able to utilize nitrate for the oxidation of methane. Methane is one of the most common carbon sources in the Fennoscandian deep subsurface. It is synthesized both biotically and abiotically, and distinguishing between the two is difficult in deep subsurface environments (Kietäväinen and Purkamo). The versatility of ways for biological methanogenesis in deep subsurface and the syntrophic/co-existing networks of unusual metabolisms involved in this process is fascinating. Ancient carbon stored in the bedrock may be recycled by fermenters in to building blocks for methane or other compounds, which makes the isotopic determination of methane as biotic or abiotic challenging.

## Conclusions

The deep biosphere has the potential to reveal novel factors about the evolution of life on Earth. There is still much to learn about the size of the microbial communities, the genetic variation and metabolic functions in the deepest part of the biosphere. For example, microorganisms tend to attach to surfaces forming biofilms, thus altering the impact of the microorganisms in deep subsurface environments, and these biofilms with their impact on the whole microbial community are yet near impossible to replicate in the laboratory. We also get a good idea only of the microorganisms resembling already known ones, leaving much of our obtained sequence data as part of the dark matter. In order to find out how the deep subsurface microbial communities really work and interact with their biotic and abiotic environment and their impact on globally important phenomena, such as climate change, we need to be able to study them *in situ* as well as improve the databases against which we compare our sequence data. These are some of the biggest challenges the deep biosphere research community faces to date.

## Author contributions

MB (lead editor) compiled the editorial and wrote the paper with assistance from LA.

### Conflict of interest statement

The authors declare that the research was conducted in the absence of any commercial or financial relationships that could be construed as a potential conflict of interest.
